# The Efficacy of Bamboo Charcoal in Comparison with Smectite to Reduce the Detrimental Effect of Aflatoxin B1 on *In Vitro* Rumen Fermentation of a Hay-Rich Feed Mixture

**DOI:** 10.3390/toxins6072008

**Published:** 2014-07-10

**Authors:** Ya-Hui Jiang, Ping Wang, Hong-Jian Yang, Ying Chen

**Affiliations:** 1 State Key Laboratory of Animal Nutrition, College of Animal Science and Technology, China Agricultural University, Beijing 100193, China; E-Mail: jiangyahui.ff@163.com; 2 Agro-product Safety Research Centre, Chinese Academy of Inspection and Quarantine, Beijing 100123, China; E-Mails: wangp_129@163.com (P.W.); chenyingcaiq@163.com (Y.C.)

**Keywords:** bamboo charcoal, smectite clay, aflatoxin B1, rumen fermentation, *in vitro*

## Abstract

Two commercial materials, a bamboo charcoal (BC) and a smectite clay (SC), were assessed *in vitro* with aflatoxin B1 (AFB1) in an equilibrium adsorption test. The adsorption capacity and proportion adsorbed (0.381 μg/mg, 0.955) for BC were greater than for SC (0.372 μg/mg, 0.931). The effects of *in vitro* ruminal fermentation of hay-rich feed incubated with 1.0 μg/mL AFB1 for 0–10 g/L doses of BC and SC were measured at 39 °C for 72 h. The BC and SC binders increased AFB1 loss at dosages ≥1.0 g/L (*p* < 0.0001). Average AFB1 loss (*p* < 0.0001) was greater for SC (0.904) than BC (0.881). Both SC and SC addition increased *in vitro* dry matter loss, and the average dry matter losses were similar. Asymptotic gas volume and volatile fatty acid production were greater for BC than for SC (*p* < 0.0001). Thus, BC may be as effective as SC in removing aflatoxin B1’s detrimental effects on rumen degradability and fermentation under the occurrence of microbial aflatoxin degradation.

## 1. Introduction

Aflatoxin B1 (AFB1), a toxin produced by *Aspergillus flavus* or *Aspergillus parasiticus* fungi, is highly mutagenic and carcinogenic to animals [1]. The transfer rates from dietary AFB1 to aflatoxin M1 in milk have been reported to be 0.01–0.02 [2], and the carry over rate is positively related with the milk yield when large amounts of contaminated concentrate are consumed [3]. Aflatoxin contaminants in feeds fed to ruminants are more problematic than other mycotoxins, because they are only partially degraded by rumen microorganisms [4]. Over the years, various methods, including chemical (e.g., ammoniation, alkalization), physical (e.g., activated charcoal, bentonite and hydrated sodium calcium aluminosilicate (HSCAS)) and biological (e.g., bacteria, yeasts, fungi and enzymes) processes, have been proposed and tested as tools to overcome AFB1 contamination in food and feeds [5]. Among the physical methods to control mycotoxin toxicity, smectite-containing products are made from naturally variable montmorillonite or bentonite clays that have high *in vitro* aflatoxin adsorption capacity and are wildly recommended, though the data of the demonstrated *in vivo* product efficacy have been limited until now [6,7]. Bamboo is a renewable, readily available forest resource in China and many other Asian countries. It can be harvested with little damage to sensitive ecosystems, due to its rapid growth rate (1–4 cm/h during the fertile period). The many pores make bamboo charcoal an excellent adsorbent, and it has been widely used in, e.g., medicines, cosmetics and food processing [8]. However, no literature has been found that has assessed the adsorption capacity of bamboo charcoal as an alternative sequestering agent for binding mycotoxins.

The high cost and health risk of the *in vivo* testing of aflatoxin enterosorbents in farm animals calls for valid *in vitro* methods for the selection of potentially useful sorbents for subsequent *in vivo* studies. Several *in vitro* methods have been developed to study AFB1 binding by sorbents. For instance, Lemke *et al*. [9] reported a multi-tiered approach that simulated the digestive process in non-ruminants, and Spotti *et al.* [10] developed a very low-cost, simple and rapid way to evaluate the *in vitro* adsorptive ability of a binder in ruminant animals. The authors’ previous study noted that the *in vitro* rumen microbial activity declined with the increase of the AFB1 dosage in cultural fluids [11]. The effect was more pronounced for a hay-rich diet than for a maize-rich diet. The applicability of different binders is to bind dietary mycotoxins and reduce their absorption in the gastrointestinal tract of animals [12]. In the present study, a smectite clay product was chosen as an aluminosilicate containing reference binder with a high adsorbing capacity for aflatoxin [6,7], and the objective was to compare bamboo charcoal with the selected smectite clay with respect to AFB1 adsorption capacity and the ability to reduce the detrimental effects of AFB1 on rumen fermentation.

## 2. Methods

### 2.1. Binders

A bamboo charcoal (BC) was gifted by the Suichang Biyan Bamboo Charcoal Company Ltd. (Suichang, Zhejiang, China). The quality-labeled product contained 850–880 g/kg carbon and 20–40 g/kg ash, as indicated by the manufacturer. The selected smectite clay (SC) was a commercial product named ConditionAde™200HPC (Oil-Dri Co. Ltd., Chicago, IL, USA), and it contained 450–650 g/kg of smectite, as guaranteed by the product. All of these products were dried at 65 °C for 24 h and ground to pass a 2.0-mm sieve, and their density, specific surface area and pore volume are in Table 1.

**Table 1:** Physical characteristics of two quality-labeled binders used in the Experiments 1 and 2.

Binder	Density (kg/m^3^)	Surface area (m^2^/g)	Pore volume (cm^3^/g)
Smectite clay	618	115	0.296
Bamboo charcoal	800	300	0.300

In Experiment 1, an *in vitro* AFB1 binding equilibrium test was conducted to determine the adsorption capacity and proportion of each binder, assuming that the amount of binder relative to toxin is not a limiting factor. Since demonstrated significant *in vitro* binding capacity might not correlate directly with significant biological efficacy, batch cultures to mimic the rumen environment in Experiment 2 were done to determine the efficacy of BC in comparison with SC to reduce the detrimental effect of AFB1 on the rumen fermentation of a hay-rich feed mixture.

### 2.2. Adsorption Capacity and Adsorption Proportion of Two Binders for the Binding of Aflatoxin B1 (Experiment 1)

#### 2.2.1. Experimental Design

Under the presence of 4 mg/L AFB1 in culture fluids, BC and SC with the addition level of 10 g/L in a buffer (pH 6.85) [13] were assessed *in vitro* for their adsorption capacity and adsorption proportion for the mycotoxin after 3, 6, 12, 24, 48 and 72 h incubation. Within each binder, there were five replicates for each incubation time.

#### 2.2.2. *In Vitro* Incubation and Sampling Procedure

The buffer was freshly prepared, bubbled and saturate with CO_2_ until the pH reached 6.85 prior to the incubation. Following the aflatoxin binding equilibrium test method of Vekiru *et al*. [14], 50 mg of SC or BC binders were individually weighed into 10-mL volume culture tubes containing 5.0 mL of the buffer. Afterwards, 0.1 mL of a working solution of AFB1 (Alexis Corporation, San Diego, CA, USA) dissolved in methanol were added to the tubes, resulting in final concentrations of 4 μg/mL AFB1 and 10 g/L binder, in accordance with the reference levels [15]. Meanwhile, 0.1 mL methanol were added to AFB1-free blank tubes with the addition of BC or SC. The whole experiment was completed in two batch cultures at a one day interval. The tubes incubated at 39 °C were gently agitated and removed at 3, 12 and 48 h in the first batch, and the rest of the tubes were done at 6, 24 and 72 h in the second batch. After the removal, the tubes were vortexed and immediately centrifuged at 10,000× *g* for 10 min at room temperature, and the supernatants were collected. The pellets were resuspended two times in 5 mL methanol by vortexing for 30 s and shaking at 39 °C for 1 h. All three supernatants were pooled together for later AFB1 analyses to calculate the adsorption capacity and proportion.

### 2.3. Animals and Rumen Fluid Collection

Three lactating multiparous Holstein cows, fitted with ruminal cannulas (Type 2C: Bar Diamond Inc., Parma, ID, USA), served as donor animals for rumen fluid collection in the later Experiment 2. The cows were fed daily 40 kg of a total mixed ration (Table 2) with a moisture content of 480 g/kg as fed, and the ration was divided into 2 equal portions offered at 06:00 and 18:00. The rumen fluids (700 mL), collected from each animal 2 h after the morning feeding, were squeezed through four layers of cheesecloth and mixed in equal proportion. The mixed rumen fluid was then transferred into a thermos pre-warmed at 39 °C and served as the inocula for the later batch culture.

**Table 2:** Ingredients and chemical composition of the ration fed to cows.

Items	Value
*Ingredients (g/kg DM)*	
Corn silage	250
Chinese wildrye grass hay	167
Alfalfa hay	83
Corn meal	267
Soybean meal	138
Wheat bran	69
Limestone	11
Calcium phosphate	6.1
Salt	4.4
Premix ^†^	4.5
*Nutrients*	
Net energy for lactation (MJ/kg DM)	6.69
Crude protein (g/kg DM)	160
Neutral detergent fiber (g/kg DM)	382
Acid detergent fiber (g/kg DM)	225

Note:^ †^ The trace mineral and vitamin premix contained Cu 3 g/kg, Zn 12 g/kg, Mn 4.8 g/kg, Fe 10 g/kg, Co 0.2 g/kg, I 0.1 g/kg, Se 0.1 g/kg, vitamin A 1000 IU/g, vitamin D3 250 IU/g, vitamin E 10 IU/g and vitamin B3 5 mg/g.

### 2.4. Effect of BC and SC on In Vitro Rumen Fermentation of a Hay-Rich Feed in the Presence of AFB1 (Experiment 2)

#### 2.4.1. Preparation of a Hay-Rich Feed

Chinese wildrye grass (*Leymus chinensis*) hay prepared at the late-bloom stage was chopped (2–5 mm), dried at 65 °C overnight in a forced air oven and ground in a Wiley mill to pass a 2.0-mm sieve. Maize meal (2 mm), stored in the laboratory, was mixed with the chopped hay in a 1:4 ratio to make a hay-rich substrate. The chemical composition (calculated per kg DM) of the hay-rich feed was: 133 g crude protein, 565 g neutral detergent fiber, 312 g acid detergent fiber and 54 g ash.

#### 2.4.2. Experimental Design

A completely randomized block design was applied to determine the effect of BC in comparison with SC at four doses (0 (control), 0.1, 1, 10 g/L) on *in vitro* ruminal fermentation of the hay-rich substrate in the presence of 1.0 µg/mL AFB1. Meanwhile, the methanol negative controls included the substrate, but no binder or AFB1. For each treatment were arranged four fermentations, and the batch culture was repeated in three runs.

#### 2.4.3. *In Vitro* Ruminal Batch Cultures

Briefly, 500 mg of the substrate were weighed into 120-mL bottles containing 7.5, 75 or 750 mg of the binders. To each bottle were added 25 mL of the filtered rumen fluid, 49 mL of the buffer [13], as noted in Experiment 1, and 1.0 mL of the aflatoxin working solution of 75 mg AFB1 dissolved in 1 L methanol, purged with N_2_ for 5 s to remove air in the bottle’s headspace. Methanol (1.0 mL) was added to the negative control bottles with the substrate. All bottles were sealed with butyl rubber stoppers and Hungate’s screw-caps. A one-off use transfusion needle was inserted into bottles through the stopper, and its pipe was immediately connected to each gas inlet of the automated gas production recording system (AGRS, Beijing, China) [16]. All bottles were incubated at 39 °C for 72 h. Substrate-free blank bottles containing buffer, ruminal fluid and AFB1 were run simultaneously to correct difference between the runs of batch culture due to variation in rumen fluid preparation. 

#### 2.4.4. Gas Production and Curve Fitting

The cumulative gas production values (GP, mL/g dry matter), exported from the automated gas production recording system, were fitted with time (t) to the exponential model [17] as Equation (1):

*GP = b ×* [1 − e^−*c* × (*time* − *Lag*)^] (1)

where *b* is the asymptotic gas production; *c* is the gas production rate; and *t* is the gas recording time. The parameters *b*, *c* and *Lag* were estimated by an iterative least squares procedure using the NLIN procedure of the Statistical Software Package for Windows (version 9.02, 1999; SAS Institute Inc., Cary, NC, USA). The average gas production rate (AGPR, mL/h) [18] was calculated to obtain the rate between the start of the incubation and the time at which the cumulative gas production was half of its asymptotic value with Equation (2):

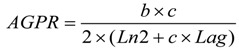
(2)


#### 2.4.5. Sampling Procedure and Digestibility Determination

After the incubation, the pH in the culture fluids was measured immediately, and the whole biomass culture (75 mL) in each bottle was removed from the bottles to 100 mL tubes and centrifuged at 10,000× *g* for 15 min at 4 °C. The supernatants were sampled later for ammonia N and volatile fatty acid (VFA) analyses and AFB1 analysis to estimate the mycotoxin disappearance rate. The pellets remaining in the tubes were resuspended in 75 mL pure methanol solution, agitated and washed at 39 °C for 1 h to partially remove AFB1, presumably not incorporated into the adsorbent-AFB1 complexes. Afterwards, the tubes were then centrifuged at 10,000× *g* for 15 min, and these supernatants from the above washes were sampled for later analysis of the residual AFB1 concentration to estimate the recovered quantity of AFB1 that would not be detoxified, but that would be bound to the hay-rich feed and binders in the system. The whole residual pellets were dried at 105 °C to a constant weight. *In vitro* dry matter disappearance (IVDMD) was calculated as the dry matter (DM) loss, represented as the difference between the original incubated DM and the residual DM, corrected by blanks [16].

### 2.5. Chemical Analysis and Calculations

Representative samples of the hay and maize meal were analyzed, respectively, following the standard method [19] for DM (ID 930.5), crude protein (ID 984.13) and ash (ID 942.05). Neutral detergent fiber and acid detergent fiber contents were analyzed [20] and expressed without residual ash.

Following the method of Upadhaya *et al.* [21], 0.3 mL of the supernatant samples in Experiments 1 and 2 were added to 1.5-mL Eppendorf tubes and mixed thoroughly with 0.7 mL methanol. The extracted AFB1 samples were immediately diluted to a final methanol concentration of 70% (v/v) with deionized water. The concentration of AFB1 in the sample extracts was determined at 490 nm with an AFB1 enzyme-linked immunoassay test kit (Brins-livePro Biotechnology Co., Ltd., Beijing, China). All chemical analyses were done in triplicate.

Ammonia N concentration in the supernatant samples resulting from the first centrifugation in Experiment 2 was measured at 637 nm [22]. The supernatant samples (1 mL) were mixed with 0.3 mL of 250 g/L meta-phosphoric acid solution for 30 min and centrifuged at 10,000× *g* for 15 min at 4 °C. The concentrations of acetate, propionate, butyrate, iso-butyrate, valerate and iso-valerate in the supernatants were measured by a gas chromatography (GC522, Wufeng Instruments, Shanghai, China). The ratio of non-glucogenic to glucogenic acids (NGR) [23] was calculated as Equation (3):

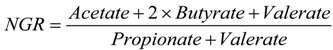
(3)
where VFAs were expressed in molar proportions of total volatile fatty acid production.

### 2.6. Statistical Analysis

Data in Experiment 1 consisted of 2 binders, 6 incubation times and 5 fermentations, making a total of 60 observations, and these were analyzed using a general linear model in which the fixed effects of binder and incubation time were considered. Data in Experiment 2 consisted of 1 control, the 3-dose level of each binder of SC and BC, 4 fermentations and 3 runs, making a total of 84 observations, and the statistical analyses were performed using a general linear model in which the fixed effects of binder and dosage were considered. All of the analyses were performed using the statistical software package for Windows (version 9.02, 1999; SAS Institute Inc., Cary, NC, USA). Least squares means and standard errors (SEM) for the measured variables were calculated. The means within each binder were compared using a multiple comparison test (Tukey), and orthogonal contrasts were used to assess the difference between SC and BC. Significance was declared at *p* < 0.05, unless otherwise noted.

## 3. Results

### 3.1. Experiment 1

**Figure 1:**
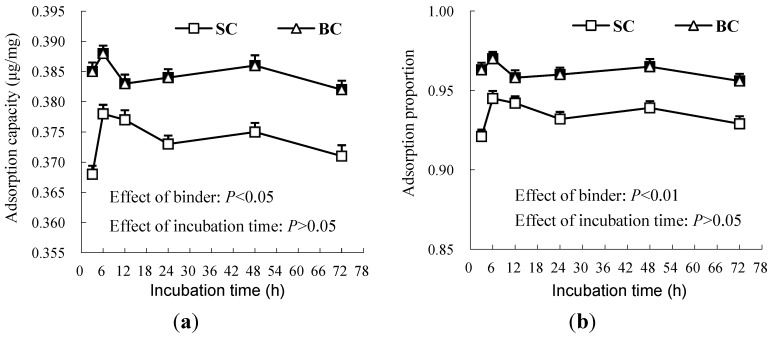
The adsorption capacity (**a**) and proportion (**b**) of smectite clay (SC) and bamboo charcoal (BC) for binding aflatoxin B1 (AFB1) *in vitro* at different incubation times (Experiment 1).

Notes: Aflatoxin adsorption capacity (a) was calculated as (*C_0_* − *C_eq_*) × *V*/*m*; and the aflatoxin adsorption proportion (b) was calculated as (*C_0_* − *C_eq_*)/*C_0_*, in which *C_0_* is the initial concentration of AFB1 (4 µg/mL) spiked in the buffer; *C_eq_* is the residual AFB1 (µg/mL) recovered from the pellets after the methanol washing and centrifuging procedure; *V* is the volume of incubation system (mL); *m* is the mass weight (mg) of each binder.

Neither the adsorption capacity nor the proportion differed in response to incubation time (Figure 1), but they were comparatively higher in BC than in SC.

### 3.2. Experiment 2

As shown in Table 3, IVDMD increased against the addition of SC (*p* = 0.048) and BC (*p* < 0.0001), but no differences occurred for IVDMD between two binders (Table 2). Cumulative gas production at 72 h (*p* < 0.05) and asymptotic gas production (*p* < 0.0001) increased when a concentration greater than or equal to 1.0 g/L was applied for both binders. The asymptote was greater in BC than SC treatments (*p* < 0.0001). The parameter c value was decreased by the SC dosage of 10 g/L (*p* = 0.0006) and the high BC dosages (≥1 g/L, *p* < 0.0001), and it was lower in the BC than in the SC treatments (*p* < 0.0001). Both SC (*p* = 0.0001) and BC (*p* < 0.0001) additions decreased the fermentation lag time. The addition of SC or BC did not alter AGPR values compared to the value in the control. No differences in lag time and AGPR were observed between two binders.

AFB1 disappearance (Table 4) was increased by the addition of SC or BC at a dose level ≥1.0 g/L (*p* < 0.0001), and it was greater in SC than BC (*p* < 0.0001). Neither SC nor BC treatments altered AFB1 recovery, but the recovery value was greater in BC than SC addition treatments (*p* = 0.035).

**Table 3:** The effect of different doses of bamboo charcoal in comparison with smectite clay on *in vitro* dry matter disappearance (IVDMD) and gas production (GP) of a hay-rich feed in the presence of 1.0 µg/mL aflatoxin B1 (Experiment 2).

Items	NC ^*^	Smectite clay				SEM ^‡^	*p*-value	Bamboo charcoal				SEM ^‡^	*p*-value	Contrast ^§^
		Control ^†^	0.1 g/L	1 g/L	10 g/L			Control ^†^	0.1 g/L	1 g/L	10 g/L			
IVDMD (g/kg DM)	604	550 ^b^	586 ^a,b^	593 ^a^	599 ^a^	11.9	0.048	550 ^b^	576 ^a,b^	589 ^a^	594 ^a^	7.8	<0.0001	0.184
GP at 72 h (mL/g DM)	208.4	147.0 ^b^	151.0 ^b^	169.1 ^a,b^	183.2 ^a^	6.86	0.021	147.0 ^b^	148.0 ^b^	179.3 ^a^	193.2 ^a^	6.20	0.0014	0.366
*Fermentation kinetics* ^#^														
GP_max_ (mL/g DM)	209.1	186.7 ^c^	193.8 ^c^	240.4 ^b^	282.9 ^a^	6.60	<0.0001	186.7 ^c^	182.5 ^c^	270.2 ^b^	315.9 ^a^	10.77	<0.0001	<0.0001
c (/h)	0.085	0.021 ^a^	0.020 ^a^	0.020 ^a^	0.012 ^b^	0.0010	0.0006	0.021 ^a^	0.020 ^a^	0.015 ^b^	0.007 ^c^	0.0009	<0.0001	0.001
Lag time (h)	0.008	0.021	0.020	0.018	0.010	0.0007	0.0001	0.021	0.020	0.015	0.007	0.0010	<0.0001	0.458
AGPR (mL/h)	3.92	2.67	2.82	2.84	2.85	0.197	0.640	2.67	2.88	2.95	2.73	0.153	0.641	0.711

Notes: ^a,b,c ^Means in a row without a common superscript letter differ within a subclass as the noted *p*-value; NC ^*^ negative control fermentation of the hay-rich substrate without the inclusion of binder and aflatoxin B1; ^†^ fermentation of the hay-rich substrate without binder inclusion; ^‡^ standard error of least squares means; ^§^ statistical *p-*value estimated for the comparison between smectite clay and bamboo charcoal; ^#^ the nonlinear equation [17], GP (mL/g DM) = GP_max_ × [1 − e^−c × (time − Lag)^], was used to analyze the gas production kinetic data. GP_max_, asymptotic gas production; c, gas production rate; Lag, lag phase before gas production commenced; AGPR, average gas production rate when half of the asymptotic gas volume was produced.

**Table 4:** The effect of different doses of bamboo charcoal in comparison with smectite clay on aflatoxin B1 (AFB1) disappearance, mycotoxin recovery and fermentation characteristics in the cultures after a 72-h *in vitro* incubation of a hay-rich feed in the presence of 1.0 µg/mL AFB1 (Experiment 2). VFA, volatile fatty acid.

Items	NC ^*^	Smectite clay				SEM ^‡^	*p-*value	Bamboo charcoal				SEM ^‡^	*p-*value	Contrast ^§^
		Control ^†^	0.1 g/L	1 g/L	10 g/L			Control ^†^	0.1 g/L	1 g/L	10 g/L			
AFB1 disappearance (µg/µg)	-	0.836 ^c^	0.844 ^c^	0.901 ^b^	0.969 ^a^	0.0073	<0.0001	0.836 ^b,c^	0.818^c^	0.862 ^b^	0.962 ^a^	0.0092	<0.0001	<0.0001
AFB1 recovery (µg/µg)	-	0.062	0.059	0.042	0.026	0.0112	0.186	0.062	0.068	0.055	0.043	0.0099	0.197	0.035
Final pH	6.76	6.88	6.88	6.88	6.82	0.023	0.215	6.88	6.93	6.99	6.87	0.053	0.445	0.081
Ammonia N (mM)	15.7	13.6	13.6	13.1	13.2	0.47	0.816	13.6	14.1	13.7	13.2	0.31	0.332	0.257
Total VFA ^#^ (mM)	78.7	68.0	68.5	66.2	60.4	3.64	0.487	68.0 ^b^	81.3 ^a^	85.8 ^a^	87.1 ^a^	2.50	0.012	<0.0001
Acetate (mol/100 mol)	71.8	70.2 ^a^	68.7 ^a,b^	68.0 ^b^	67.2 ^b^	0.59	0.025	70.2	70.3	69.0	68.8	0.57	0.184	0.001
Propionate (mol/100 mol)	18.5	20.0 ^c^	21.7 ^b^	22.1 ^a,b^	22.5 ^a^	0.19	<0.0001	20.0 ^b^	20.4 ^b^	21.5 ^a^	22.1 ^a^	0.24	0.0004	0.0004
Butyrate (mol/100 mol)	4.09	4.19	4.31	4.36	4.40	0.13	0.700	4.19	4.03	4.24	3.98	0.10	0.322	0.002
Iso-butyrate (mol/100 mol)	0.74	0.71	0.78	0.73	0.82	0.026	0.227	0.71	0.75	0.76	0.76	0.020	0.974	0.263
Valerate (mol/100 mol)	1.32	1.49	1.51	1.64	1.57	0.056	0.403	1.49	1.47	1.46	1.40	0.049	0.709	0.007
Iso-valerate (mol/100 mol)	2.96	2.97	3.06	2.99	3.07	0.010	0.916	2.97	2.97	3.02	2.92	0.074	0.817	0.286
NGR ^ζ^	3.97	3.76 ^a^	3.40 ^b^	3.29 ^b^	3.22 ^b^	0.039	0.003	3.76 ^a^	3.65 ^a,b^	3.44 ^b,c^	3.32 ^c^	0.055	0.006	0.001

Notes: ^a,b,c ^Means in a row without a common superscript letter differ within a subclass as the noted *p*-value; NC ^*^ negative control fermentation of the hay-rich substrate without the inclusion of binder and AFB1; ^†^ fermentation of the hay-rich substrate without binder inclusion; ^‡^ standard error of least square means; ^§^ significant *p*-value for the comparison between smectite clay and bamboo charcoal; ^#^ total concentration of acetate, propionate, butyrate, iso-butyrate, valerate and iso-valerate; ^ζ^ ratio of non-glucogenic to glucogenic acids.

The pH and ammonia N levels did not differ between binders and between dosages (Table 4). The total VFA concentration was lower in SC than BC (*p* < 0.0001). The SC addition numerically decreased the total VFA concentration, whereas the BC addition treatments increased the total VFA concentration (*p* = 0.012). No differences in response to the addition of SC or BC occurred for molar proportions of butyrate, iso-butyrate, valerate or iso-valerate. The molar acetate proportion was decreased by SC compared to the control (*p* = 0.025), but it did not differ among BC additions. The molar acetate proportion was greater in BC than SC addition (*p* = 0.001). The molar propionate proportion was increased by both SC (*p* < 0.0001) and BC (*p* = 0.0001), and it was greater in SC than BC (*p* = 0.0004). Molar proportions of butyrate (*p* = 0.002) and valerate (*p* = 0.007) were lower in BC than SC. Consequently, the NGR value was decreased by the addition of SC (*p* = 0.003) or BC (*p* = 0.006), and it was greater in BC in comparison with SC (*p* = 0.001).

## 4. Discussion

### 4.1. AFB1 Adsorption by BC in Comparison with SC

The adsorption capacity reflects the ability of a binder to adsorb AFB1 when the AFB1 was presented in a sufficient concentration. Physical methods are considered to be the most efficient way to reduce the toxicity of AFB1, in which smectite clays have been evaluated for their binding capacity and affinity for AFB1 [24,25,26,27]. The surface area, sodium:calcium ratio, porosity characteristics, the amount of smectite, the cation exchange capacity, the hydrated radius of the interlayer cations, the occurrence of Fe and/or Mg in the smectite structure, the amount of organic carbon and the hydrophobicity of the smectite surface in the clays may be involved in a complex way to bind aflatoxin [6,27,28,29,30]. The *in vivo* binding capacity of activated charcoal in different sources varied due to the variations in the surface area, although all of the activated charcoal bound over 95% of the aflatoxin *in vitro* [6]. Hydrated sodium aluminosilicate was regarded as one of the most widely used binders for AFB1 adsorption, and it exhibited strong AFB1-binding ability *in vitro* and *in vivo*. Duarte *et al.* [31] noted that the *in vitro* AFB1 binding capacity values of all HSCAS sequestering agents were greater than 95%. Such high AFB1 binding capacity was observed for SC at a concentration of 10 g/L. Although activated charcoal was also widely used for its adsorption capacity and adsorption proportion for mycotoxin, the practical value of activated carbon as a feed additive might be limited by the fact that it also binds other nutrients, such as vitamins [32]. BC had a good capability for adsorbing dyes in wastewater, because it had larger pores than activated charcoal, allowing it to adsorb larger molecules [33]. In the present study, the binding ability of BC for AFB1 reached an equilibrium state after 3 h, and it remained stable throughout the rest of the incubation period, with the same adsorption pattern as seen for SC. The overall AFB1 adsorption capacity and proportion were comparatively greater in BC than SC (Figure 1), suggesting that BC could be as effective as SC in adsorbing AFB1. 

The adsorption proportion reflects the mycotoxin adsorption ability of a binder when the addition level of the binder is not limited, and its value is affected by the binder pore size and surface area, as well as the mycotoxin structure and concentration. The *in vitro* adsorption proportion of BC (average value = 0.955) in the present study (Figure 1) was remarkably greater than 0.673, noted for the yeast cell extracts [34], and similar to the *in vitro* result of HSCAS [31], the adsorption capacity for HSCAS, which was also studied in an *in vivo* experiment, shows a higher binding capacity for AFB1 in feed and can reduce the AFB1 transmission from feed into milk [7]. The surface area and the water holding capacity are important factors affecting the binding capacity for AFB1. Regarding these factors in the above, BC maybe show a high binding capacity for AFB1 an *in vivo* experiment, because of the large surface area, the many pores and a high water holding [33], but the *in vivo* study results should be measured following *in vitro* evaluation to clarify if BC does or does not do not bind vitamins or minerals, otherwise the use of this absorbent in animal nutrition could be limited or not recommended.

### 4.2. Disappearance of AFB1 in the Presence of BC in Comparison with SC

Spotti *et al.* [10] developed an *in vitro* method to test the ability of sorbent materials to bind aflatoxin in 1 mL ruminal fluids incubated for 2 h at 39 °C. With the application of these methods, the binders containing HSCAS, SC and clinoptilotile were proven to have different adsorption characteristics for binding AFB1, though the length of exposure to AFB1 might not be adequate to mimic rumen fermentation. 

In the present study, batch cultures in Experiment 2 were conducted in a 75-mL (rumen fluid: buffer = 1:2) ruminal system for *in vitro* mimicry of 72-h rumen fermentation, and the mycotoxin disappearance rate for AFB1 was 0.836 in the control without the addition of any binders (Table 4). Engel and Hagemeister [35] reported that 42% of AFB1 was degraded when incubated *in vitro* with rumen fluid. Westlake *et al.* [36] in their *in vitro* mycotoxin studies with rumen fluid reported that the degradation of AFB1 after 12 h was <10% when added at levels of 1.0 and 10 µg/mL Forty-five percent of AFB1 was degraded when AFB1 at an initial concentration of 0.2 µg/mL was incubated at 39 °C in a 1-mL fresh ruminal fluid system for 2 h [10]. All of these reported AFB1 disappearance rates were far lower than the value of 0.836 in the present study. These results imply that the ability of rumen microorganisms to reduce AFB1 toxicity might depend on the length of time of mycotoxin exposure for rumen microorganisms. The type of microbes in the rumen influenced by the species of animal and the types of forage fed for the animal would also affect the extent of the degradation of AFB1 [21]. Kiessling *et al.* [37] found that AFB1 and ochratoxin A were not well metabolized by whole rumen fluid, although mycotoxins zearalenone, T-2 toxin, diacetoxyscirpenol and deoxynivalenol were well metabolized. Westlake *et al.* [36] reported that *Butyrivibrio fibrisolvens* was able to degrade mycotoxins zearalenone, T-2 toxin, diacetoxyscirpenol and deoxynivalenol, but not AFB1. The results in the above suggested that the mycotoxin might disturb the growth and metabolic activity of rumen microorganisms, though the microbial population of the rumen plays a role in detoxification.

In the present study, AFB1 disappearance was increased by both BC and SC addition treatments with dosages greater than or equal to 1.0 g/L compared to the control (Table 4). The average AFB1 disappearance was comparatively lower in the BC than in the SC addition treatment (0.881 *vs*. 0.904, *p* < 0.0001). The amount of AFB1, recovered from the pellet after the methanol washing procedure at 39 °C for 1 h, could reflect the quantity of AFB1 that was not destroyed by rumen microorganisms, but had that been bound to the feed and binders in the system. Both the BC and SC addition treatments resulted in low AFB1 recovery values, especially for these binders added at 10 g/L (Table 4), but comparatively higher AFB1 recovery values were observed for BC in comparison with SC (*p* = 0.035), suggesting that the optimal binder dosage to reduce the detrimental effects of aflatoxin was different for BC compared to SC. 

### 4.3. In Vitro Ruminal Fermentation Responses to BC in Comparison with SC

The *in vivo* digestibility of animal feeds can be estimated by measuring the *in vitro* gas production of feed samples incubated in ruminal fluid [17]. Westlake *et al.* [36] noted that AFB1 inhibited microbial digestion of lucerne hay incubated with bacterial, protozoal and ovine ruminal fluid preparations. In the present study, both SC and BC addition treatments increased IVDMD, cumulative gas production at 72 h and asymptotic gas production (Table 3), suggesting that both binders indeed had the ability to reduce the detrimental effects of AFB1 on microbial digestion. The addition of binder reduced the lag time of the fermentation, and asymptotic gas production was greater in the BC than in the SC addition treatments, suggesting that BC could be more effective for reducing the detrimental effect of AFB1 on rumen fermentation than SC, due to the larger pore size and the greater number of pores in BC compared to SC. 

In the present study, binder dosage did not alter ammonia N concentration, and no differences were observed for ammonia N concentration between SC and BC. In the rumen, the feed protein is usually hydrolyzed and deaminated, forming peptides and free ammonia by the rumen microorganisms [38]. If the digestion and metabolism of feed protein were inhibited by AFB1, the concentration of free ammonia would be decreased. In the present study, then, ammonia N concentrations in both SC and BC were lower than the control without any inclusions of AFB1 and binder, suggesting that the method of hydrolysis or deamination of the protein was decreased by the presence of AFB1 regardless of the binder added. A decrease of ammonia production was also observed for AFB1 ingestion at 0.2–0.8 mg/kg body weight in acute bovine aflatoxicosis [39]. 

The production of VFAs can account for over two-thirds of the energy intake in a host ruminant animal [40], and therefore, VFAs, resulting from rumen fermentation, can be regarded as an important index for fermentation efficiency. In the literature, no differences of total VFA concentration in the rumen were observed in growing lambs [41] fed 2.5 mg AFB1 per kg diet, steers [38] fed 60–600 µg/kg diet and lactating goats [42] daily fed 0.714 µmol AFB1. On the contrary, VFA production was decreased by AFB1 at 0.2–0.8 mg/kg body weight in acute bovine aflatoxicosis [39]. Regardless of the binder added, the total VFA concentration was lower in both the SC and BC groups than in the control without any inclusions of AFB1 and binder, suggesting that AFB1 present in the culture fluids decreased the growth and metabolic activity of rumen microorganisms. With the increase of the binder dose, the total VFA concentration markedly increased in the BC treatment, while it numerically decreased in SC, suggesting that BC indeed reduced the adverse effects of AFB1 on rumen microbial activity. 

The balance between the supplies of glucogenic relative to non-glucogenic fatty acids influences the efficiency of VFA utilization for different productive purposes in ruminant animals. The observed VFA pattern and the occurrence of high NGR values for BC addition treatments (Table 4) imply that the addition of BC in comparison with SC would result in greater stimulation of the production of glucogenic acids (especially propionate) in the rumen as precursor nutrients for the host animal.

## 5. Conclusions

An equilibrium mycotoxin adsorption test showed that bamboo charcoal had a comparatively higher adsorption capacity for aflatoxin B1 (AFB1) than a smectite clay. Relative to the smectite, bamboo charcoal increased feed digestibility, gas and volatile fatty acid production, which reduced the detrimental effects of AFB1 on rumen fermentation. This study compared bamboo charcoal and a smectite in sequestering AFB1 and demonstrated that bamboo charcoal can bind AFB1 as effectively as smectite under the occurrence of microbial aflatoxin degradation. 
